# Patient derived renal cell carcinoma xenografts exhibit distinct sensitivity patterns in response to antiangiogenic therapy and constitute a suitable tool for biomarker development

**DOI:** 10.18632/oncotarget.25697

**Published:** 2018-07-24

**Authors:** Julia Schueler, Kerstin Klingner, Daniel Bug, Caren Zoeller, Armin Maier, Meng Dong, Kerstin Willecke, Anne-Lise Peille, Eva Steiner, Manuel Landesfeind, John A. Copland, Gabrielle M. Siegers, Axel Haferkamp, Katharina Boehm, Igor Tsaur, Meike Schneider

**Affiliations:** ^1^ Charles River Discovery Research Services Germany GmbH, Freiburg, Germany; ^2^ Department of Cancer Biology, Mayo Clinic, Jacksonville, FL, USA; ^3^ LfB – Lehrstuhl für Bildverarbeitung, RWTH Aachen University, Aachen, Germany; ^4^ Department of Urology, University Hospital Frankfurt, Goethe University, Frankfurt am Main, Germany; ^5^ Department of Urology, Medical Center Johannes Gutenberg University, Mainz, Germany; ^6^ Department of Experimental Oncology, University of Alberta, 5-142W Katz Group Centre, Edmonton, Canada; ^7^ Department of Radiation Oncology, University Hospital of Würzburg, Würzburg, Germany; ^8^ Dr. Margarete Fischer-Bosch - Institut für Klinische Pharmakologie, Stuttgart, Germany

**Keywords:** HMGB1, renal cell carcinoma, damage associated molecular pattern, bevacizumab, VEGF

## Abstract

Systemic treatment is necessary for one third of patients with renal cell carcinoma. No valid biomarker is currently available to tailor personalized therapy. In this study we established a representative panel of patient derived xenograft (PDX) mouse models from patients with renal cell carcinomas and determined serum levels of high mobility group B1 (HMGB1) protein under treatment with sunitinib, pazopanib, sorafenib, axitinib, temsirolimus and bevacizumab. Serum HMGB1 levels were significantly higher in a subset of the PDX collection, which exhibited slower tumor growth during subsequent passages than tumors with low HMGB1 serum levels. Pre-treatment PDX serum HMGB1 levels also correlated with response to systemic treatment: PDX models with high HMGB1 levels predicted response to bevacizumab. Taken together, we provide for the first time evidence that the damage associated molecular pattern biomarker HMGB1 can predict response to systemic treatment with bevacizumab. Our data support the future evaluation of HMGB1 as a predictive biomarker for bevacizumab sensitivity in patients with renal cell carcinoma.

## INTRODUCTION

Renal cell carcinoma (RCC) represents 3-5 % of all malignancies in adults [1]. The most common histologies are clear cell (ccRCC, 87.7%), papillary (pRCC, 9.7%), and chromophobe carcinomas (cRCC, 2.5%) [2], and RCC accounts for 80% of all kidney cancers [3]. One third of these patients need systemic therapy due to development of synchronous or metachronous metastases [4]. Although recent advances in targeting the vascular endothelial growth factor (VEGF) pathway, mammalian target of rapamycin [3], and the advent of immune checkpoint inhibitors [5] have changed the therapeutic landscape for this disease, patient survival remains limited. Clinical prognostic factors, according to the International Metastatic RCC Database Consortium (IMDC), including e.g. the Karnofsky performance status and hemoglobin levels, are currently used to stratify patients into risk groups in order to select first or second line systemic treatments [6]. The duration of response and survival benefit of therapy varies considerably among patients. This might be at least partially attributed to their broad genomic heterogeneity, which leads to a highly diverse sensitivity of individual tumors to therapeutic agents [7].

Over 90% of sporadic ccRCC harbor a mutation in the Van-Hippel-Lindau protein (pVHL) which leads to the loss-of-function of the protein. [8] pVHL targets the family of hypoxia inducible factors (HIFs) for destruction so that loss of pVHL function in cells leads to constitutive upregulation of HIF1α and HIF2α. HIFs transactivate genes involved in angiogenesis, erythropoiesis, cell proliferation, and metastasis and typical HIF target genes include VEGF, erythropoietin (EPO), and angiopoietin 1 [9, 10]. In addition to the upregulation of hypoxia inducible factors due to enhanced VHL-HIF signaling pathway RCC also exhibit tumor hypoxia due to low oxygen pressure within the tumor. In general HIF1a is commonly used to indicate hypoxia in the tumor tissue and VEGF is continuously expressed throughout the tumor life cycle [11].

Recently, a set of gene mutations was identified that could predict recurrence after curative surgical treatment [12] but as of today no specific molecular marker predicts patient responses to any of the approved therapies, rendering it difficult to make treatment choices. The development and validation of biomarkers in prospective clinical trials is needed in order to predict response to various systemic therapies and lead to advances in personalized medicine [13, 14].

High mobility group box 1 (HMGB1) protein is a damage associated molecular pattern (DAMP) protein and a key mediator of inflammation [15]. HMGB1 is secreted by immune cells such as macrophages and dendritic cells in response to infection, injury or inflammatory stimuli [16]. Moreover, HMGB1 diffuses passively through leaky membranes of necrotic cells. It is overexpressed in distinct malignancies, including RCC, and exerts its role through binding to a multitude of membrane-bound receptors including Toll-like receptors 2, -4 and receptors for advanced glycation end products (RAGE) [17–20]. Although the exact molecular function of HMGB1 in cancer is largely unknown, early studies describe circulating HMGB1 as a useful tool and biomarker for malignant tissue or monitoring therapy [21, 22].

We have established a panel of patient derived RCC PDX mouse models and measured serum HMGB1 levels in the mice before and during treatment with either sunitinib, sorafenib, pazopanib, temsirolimus or bevacizumab. Bevacizumab is a humanized monoclonal antibody that directly targets VEGF for degradation [23]. Sunitinib, sorafenib and pazopanib are multi-tyrosin-kinase inhibitors that inhibit downstream targets of the VHL-HIF signaling pathway while temsirolimus inhibits mammalian target of rapamycin. [24] We found a positive correlation between the pre-treatment serum levels of HMGB1 and response to treatment with the anti-VEGFA antibody bevacizumab. These novel findings identify a potential novel biomarker for RCC, and we propose a role for HMGB1 as a tool for therapeutic decision making in patients with metastasized RCC.

## RESULTS

### RCC PDX retain histological features of the original patient tumor and mimic the molecular landscape of the disease

We aimed to establish a cancer model platform representing patient tumors in order to validate biomarkers to inform treatment decisions when considering currently available systemic therapies.

**Figure 1:**
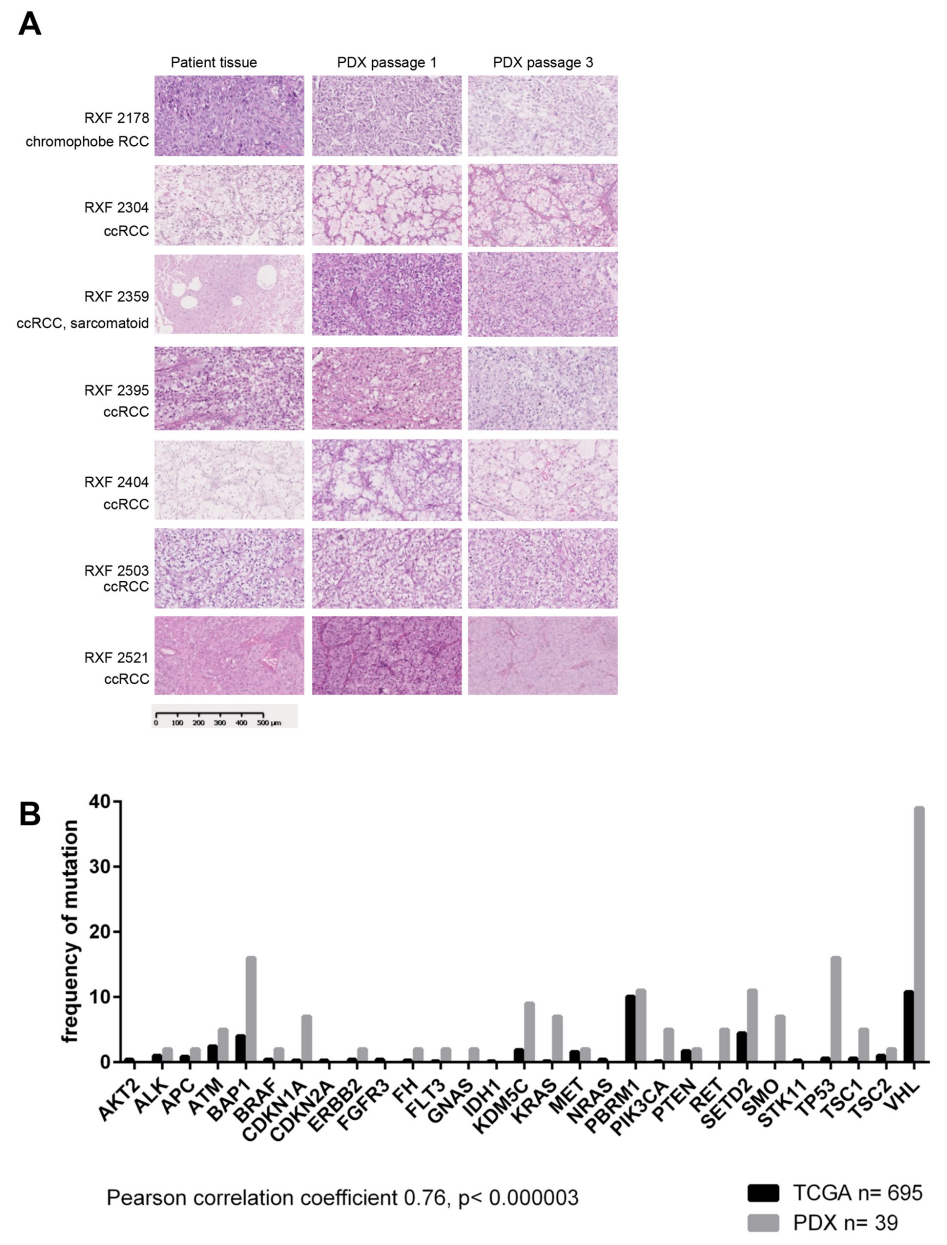
Patient derived xenografts retain histological features of the original patient tumor and mimic the molecular landscape of the disease **(A)** Histological features of selected PDX and corresponding patient tissue. H&E stains were prepared from FFPE samples of donor patient tissue as well as first and third passage of PDX derived thereof. All major histotypes of renal cancer are represented in the panel of 44 renal cancer PDX. The histological features like tumor/stroma ratio and differentiation remain stable when tumor tissue is implanted and passaged in immunocompromised mice. **(B)** 39 models were characterized by whole exome sequencing. The comparison with TCGA data revealed a significant correlation between the two data sets. Thus, our renal cancer PDX panel largely represents the molecular landscape of the human disease.

A panel of 44 PDX mouse models was generated from RCC patient tumor tissues that included 42 clear cell RCC, 1 papillary RCC and 1 chromophobe RCC. The median age of the donor patients was 61 years (range 38 – 80 years) and 42 patients did not receive any treatment prior to surgery. One patient was pretreated with sorafenib, another one with pazopanib prior to tumor resection. The established PDX models were characterized by tumor growth behavior, patho-histological examination, molecular characterization and sensitivity towards standard of care compounds (Table 1 and Supplementary Table 1). A tissue microarray (TMA) was developed that included all 44 PDX and two cell line derived RCC models (Supplementary Figure 1). The PDX models maintained their typical histopathologic appearance during serial passage in immune compromised mice, displaying the same features as the donor patient material (Figure 1A). Comparison of the molecular landscape between our own RCC PDX panel and TCGA data (TCGA Research Network: http://cancergenome.nih.gov/) of representative patient cohorts revealed that the PDX covered most of the described genetic mutations of the malignancy; relevant driver mutations including VHL, BAP1, PBRM1 and SETD2 were represented in our PDX panel. The mutational pattern of the PDX panel correlated significantly with TCGA data (Pearson correlation 0.76, p<0.000003, Figure 1B). Thus, our RCC PDX recapitulated the tumor biology of the patients´ primary tumors and the panel represents the range of RCC reported in the literature [8, 25].

**Table 1:** Characteristics of the RCC PDX panel

nr of models	pts data								PDX data				
	age, median (range)	gender (f:m)	treatment before surgery		subtype		origin of biopsy		doubling time, median (range)	SoC data available	cell line available	WES data available	gene expression data available
44	61 (38 - 80) years	11:35	none	42	KIRC	43	primary	31	6.75 (3.5 - 14.6) days	25	7	39	39
			Pazopanib	1	KIRP	1	reccurent	6					
			Sunitinib	1			metastasis	7					

### HMGB1 serum levels were significantly higher in a subset of RCCs and correlated with increased hypoxia signaling

**Figure 2:**
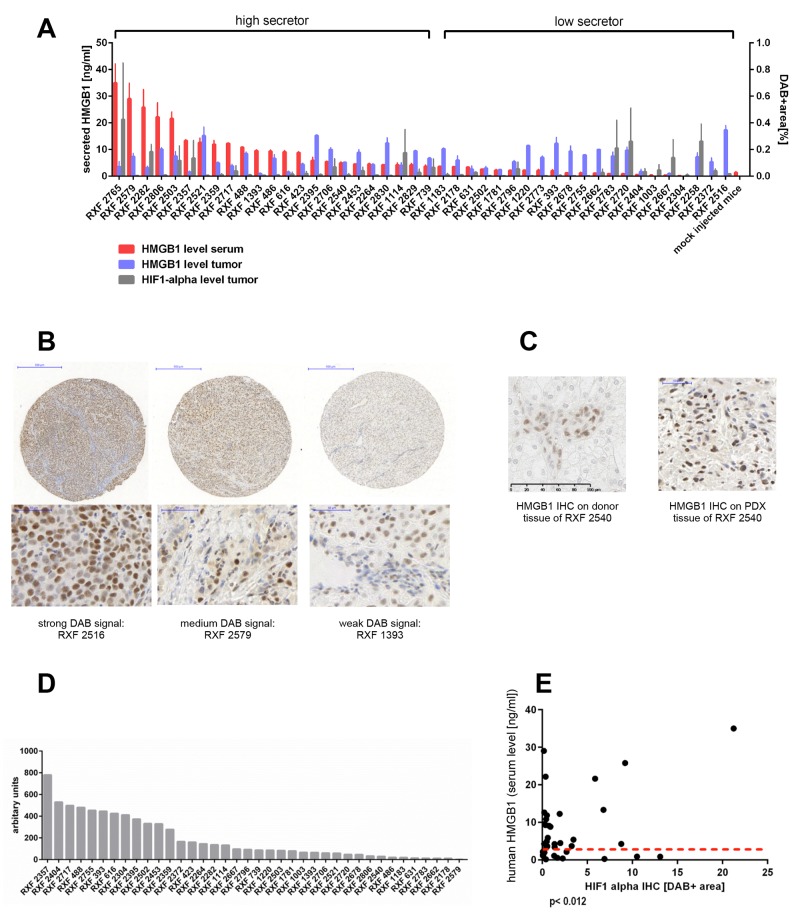
Secreted human HMGB1 levels in tumor-bearing mice do not correlate with the amount of intracellular HMGB1 or VEGF in the respective PDX but with HIF-1 alpha expression in the tumor **(A)** Serum levels of human HMGB1 were determined by ELISA in tumor bearing NMRI nude mice (n = 2-5 per model, 125 in total). Serum was taken when tumor load was between 80 mm^3^ and 250 mm^3^ prior to any treatment. Mock-injected non-tumor bearing NMRI nude mice, with matching sex and age, served as negative controls (n= 6). ELISA was performed in technical duplicates. Red bars represent mean (±SEM). The grouping in high secretors and low secretors was performed by comparing the mean HMGB1 level of an individual model with the mock-injected control. If the difference was statically significant (Mann–Whitney, two-tailed) the respective model was a high secretor. A IHC was performed for human HMGB1 or HIF-1 alpha on the renal cancer PDX specific TMA including all 44 PDX models in duplicates and two renal cancer xenografts (Caki1 and MRI-H-166). Using the OSANO software the DAB+ area for each individual TMA punch was determined and plotted as blue bars (HMGB1) or grey bars (HIF-1 alpha) representing mean (±SEM) of percentage of DAB+ area of one punch. HMGB1 as well as HIF-1 alpha expression varied markedly within the panel. **(B)** representative images of weak, median and strong DAB signal of the HMGB1 IHC. **(C)** Comparison between the donor patient and its PDX model RXF 2540 showed similar expression levels of HMGB1. **(D)** mRNA expression of human VEGFA determined by qRT-PCR on lysates of renal cancer PDX tissue. The expression level in arbitrary units is calculated as ratio of human VEGFA vs human TBP. **(E)** Serum levels of HMGB1 and level of HMGB1 determined by IHC or human VEGFA as well as murine VEGFA expression determined by qRT-PCR did not correlate. The high secretors (above the read dotted line) exhibited all different levels of IHC staining intensity and qRT-PCR expression, respectively. In contrast the HMGB1 serum levels did correlate significantly (Pearson correlation coefficient 0,39, p< 0.012) with the HIF-1 alpha IHC scoring.

We determined HMGB1 levels in the sera of tumor-bearing mice and found a distinct secretion profile. Based on the levels of secreted HMGB1, we divided the models into two different groups: low and high secretors. The latter showed mean HMGB1 levels significantly higher than the levels determined in sex and age matched non-tumor-bearing NMRI nude mice (Figure 2A, p<0.024). According to this algorithm, 23 PDX models could be allocated to the high secretor group with a mean HMGB1 level of 12.7 ± 9.6 ng/ml and 21 to the low secretor group (mean serum level of 1.8 ± 0.6 ng/ml). The cut-off value was 3.8 ng/ml, and the difference between the groups was highly significant (p< 0.0001).

In addition to HMGB1 we investigated the expression levels of VEGFA, as a main target in renal cancer treatment strategies. Furthermore, HIF-1 alpha was evaluated as key indicator for hypoxia and pseudo-hypoxia induced by pVHL loss of function.

We determined protein expression of HMGB1 and HIF-1 alpha in the RCC panel by immunohistochemistry (IHC) and their levels in the tumor xenograft panel were quantified using OSANO software. Intensity of DAB staining revealed different expression levels: RXF 2516 exemplified high expression of HMGB1 whereas RXF 1393 revealed remarkably low levels (Figure 2B, Supplementary Figure 1). Patient-matched tumor tissue showed a similar HMGB1 expression level to that of the PDX model. An example is shown for model RXF 2540 (Figure 2C).

Evaluation of murine and human VEGF by qPCR revealed variable VEGF mRNA levels within the tumor panel (Figure 2D and Supplementary Figure 2A), as was seen with HMGB1 and HIF-1 alpha protein expression. HMGB1 levels in mouse sera significantly correlated with HIF-1 alpha expression in the corresponding PDX tumor tissues. (Figure 2E, Pearson correlation coefficient 0,39, p< 0.012). In contrast, serum HMGB1 did not correlate with intracellular HMGB1 levels nor with human or mouse VEGF levels in the tumor tissues (Supplementary Figure 2B).

Mutational analysis by whole exome sequencing revealed an overrepresentation of high secreting HMGB1 models in VHL mutated RCC PDX as compared to the VHL wildtype group (Fisher exact test p<0.017). No other correlation in the mutational profile of low and high HMGB1 secretor groups could be determined (data not shown).

To evaluate serum HMGB1 levels as a possible prognostic marker, we compared overall survival data within the PDX donor patient cohort: survival of high secretor versus low HMGB1 secretors did not differ significantly (Supplementary Figure 3A). As only 25 datasets from the patient cohort could be investigated, we performed a similar analysis using TCGA data from 505 renal cancer patients. This cohort was divided by median HMGB1 gene expression and the data was adjusted for stage. Once again, no significant difference in overall survival was observed. Taken together, HMGB1 does not appear to be a prognostic marker for RCC (Supplementary Figure 3B).

### RCC PDX Tumors with high HMGB1 secretion show slower tumor growth

**Figure 3:**
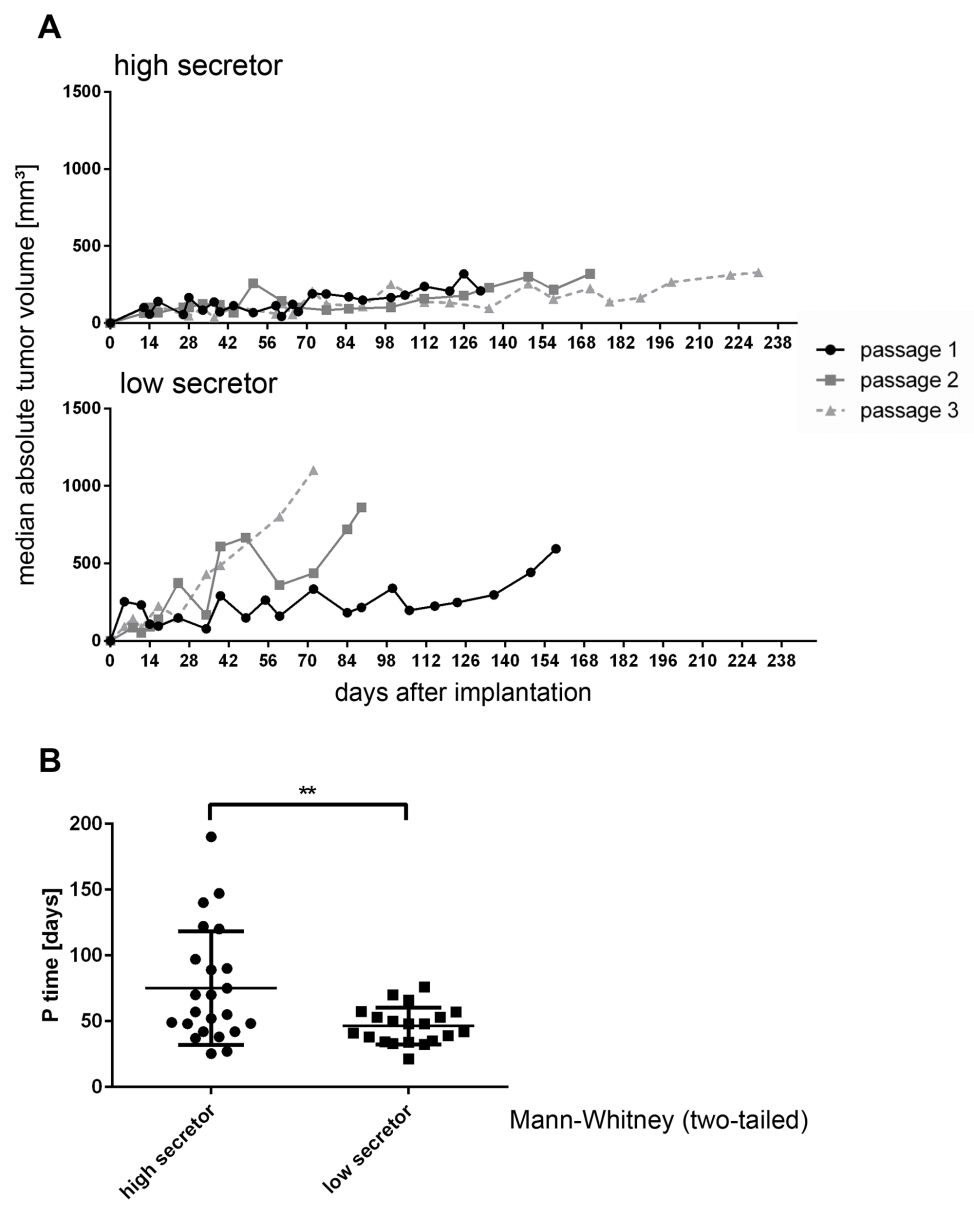
RCC PDX Tumors with high HMGB1 secretion show slower tumor growth **(A)** Tumor volume was determined by caliper measurement biweekly in individual mice over subsequent passages for all models. Each graph depicts the median volume (±SEM) of all high (upper part) and low (lower part) HMGB1 secreting tumor models in subsequent passages 1 through 3. Passage 1 was defined as tumors growing after implantation of the patient donor tissue. **(B)** The passage time, defined as time between implantation and sacrifice of the animal due to tumor burden, was determined. PDX secreting high levels of HMGB1 depicted slower tumor growth over time as models with low levels of HMGB1. This phenomenon was statistically significant when comparing the passaging times of both groups (p< 0.007, Mann–Whitney test, two-tailed).

Tumor volumes were determined in individual mice over subsequent passages for all models. Moreover the passage time, defined as the time between implantation and sacrifice of the animal due to tumor burden, was determined. Tumor growth was comparable between groups directly after implantation of the human donor material (Passage 1). The median passage time of models with high HMGB1 secretion was 109.5 ± 13.4 days vs 75.6 ± 22.0 days for the low secretor group. (Figure 3A) This difference was statistically significant (p<0.017) and became more pronounced in subsequent passages. Tumor growth rates increased over passages for the low secretor group and diminished in PDX with high HMGB1 serum levels. Comparing the passage time in days over the first three passages, PDX secreting high levels of HMGB1 had a mean passage time of 75.2 ± 22 days and the low secretor group 46.4 ± 7 days. Thus, tumors with low HMGB1 serum levels displayed distinctly more aggressive tumor growth behavior over time than tumors with high HMGB1 levels (Figure 3B, p<0.001).

### Each RCC PDX model demonstrated a specific sensitivity pattern in response to approved first and second line therapies

**Figure 4:**
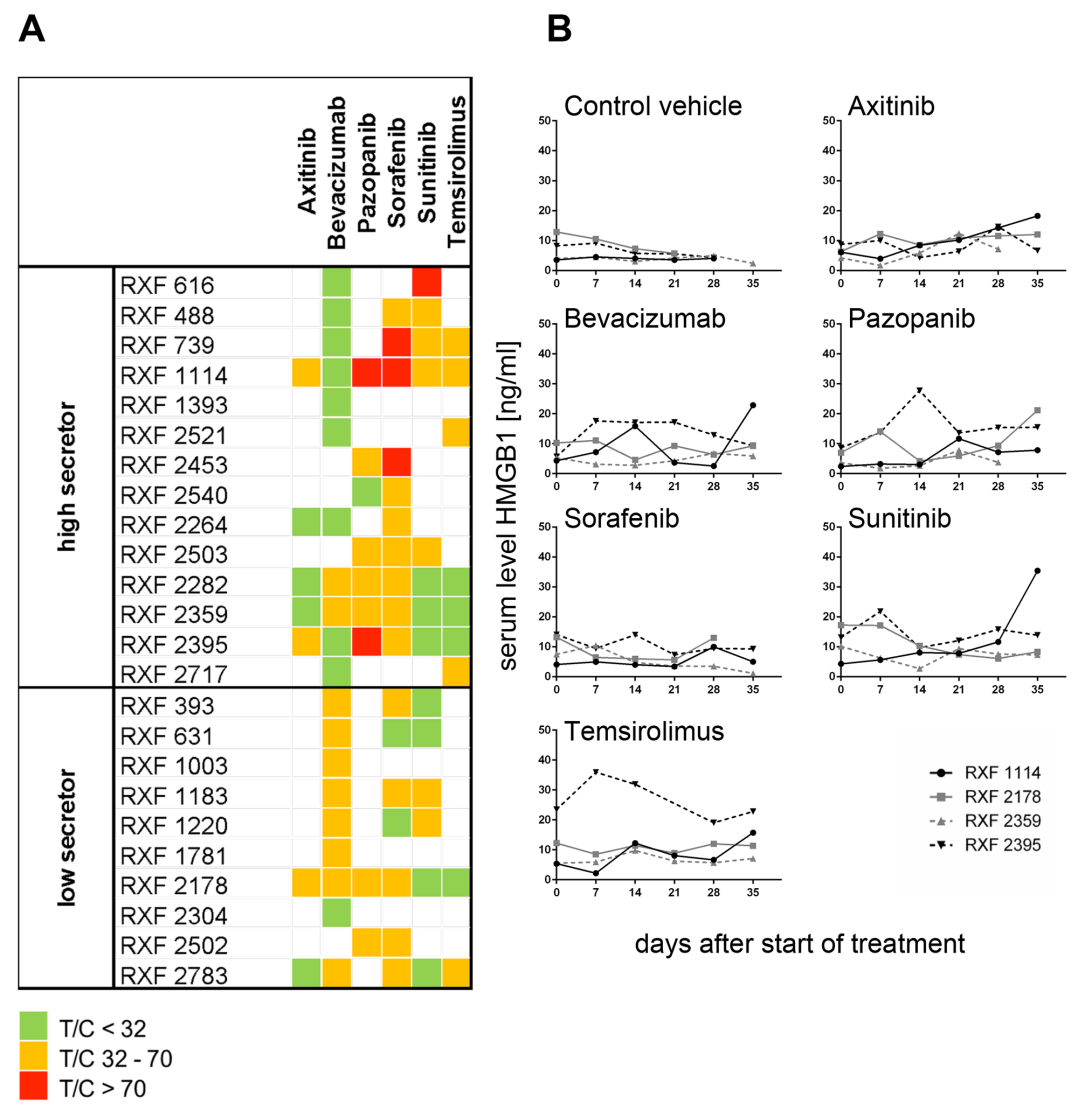
Characterization of renal cancer PDX panel by *in vivo* assessment of six standard of care compounds **(A)** Characterization of 24 renal cancer PDX by treatment with different antiangiogenic and other targeted therapies. All investigated PDX models displayed distinct sensitivity pattern against axitinib, bevacizumab, pazopanib, sunitinib, sorafenib and temsirolimus. Two – six compounds per model were tested in monotherapy. Five mice bearing bilateral tumor implants were assigned for each treatment group including the vehicle control. Optimal T/C values are plotted and categorized into highly active (green= T/C < 32%), active (orange= T/C 32 – 70%) and resistant (red = T/C > 70%). The tumor models were grouped into secreting high levels of HMGB1 (high secretor) and low levels of HMGB1 (low secretor). **(B)** In parallel, once weekly, starting one day before first treatment, serum was sampled from all mice and HMGB1 level determined by ELISA. Four representative renal cancer PDX models are shown. The treatment with different targeted compounds in different renal cancer PDX models did not affect the serum level of HMGB1.

We determined anti-tumoral activity of sunitinib, pazopanib, sorafenib, axitinib, temsirolimus and bevacizumab in 24 RCC xenografts. Every model displayed specific sensitivity to the six drugs. Overall, axitinib (mean optimal test/control (T/C) value of 27%) and temsirolimus (mean optimal T/C value of 32%) were the most active compounds; sorafenib and pazopanib showed the least efficacy (mean T/C values of 51% and 52%, respectively). Bevacizumab and sunitinib showed similar anti-tumoral activity, obtaining a mean optimal T/C value of 34% (Figure 4A). For RXF1114, RXF2178, RXF2359 and RXF2395, we determined serum HMGB1 levels one day prior to the first treatment and subsequently once weekly during treatment with the respective drug. HMGB1 levels were consistent and largely independent of the actual tumor load or treatment regimen (Figure 4B).

### Hypoxia induced secretion of HMGB1 and sensitized RCC PDX derived tumor cell lines towards treatment with bevacizumab

**Figure 5:**
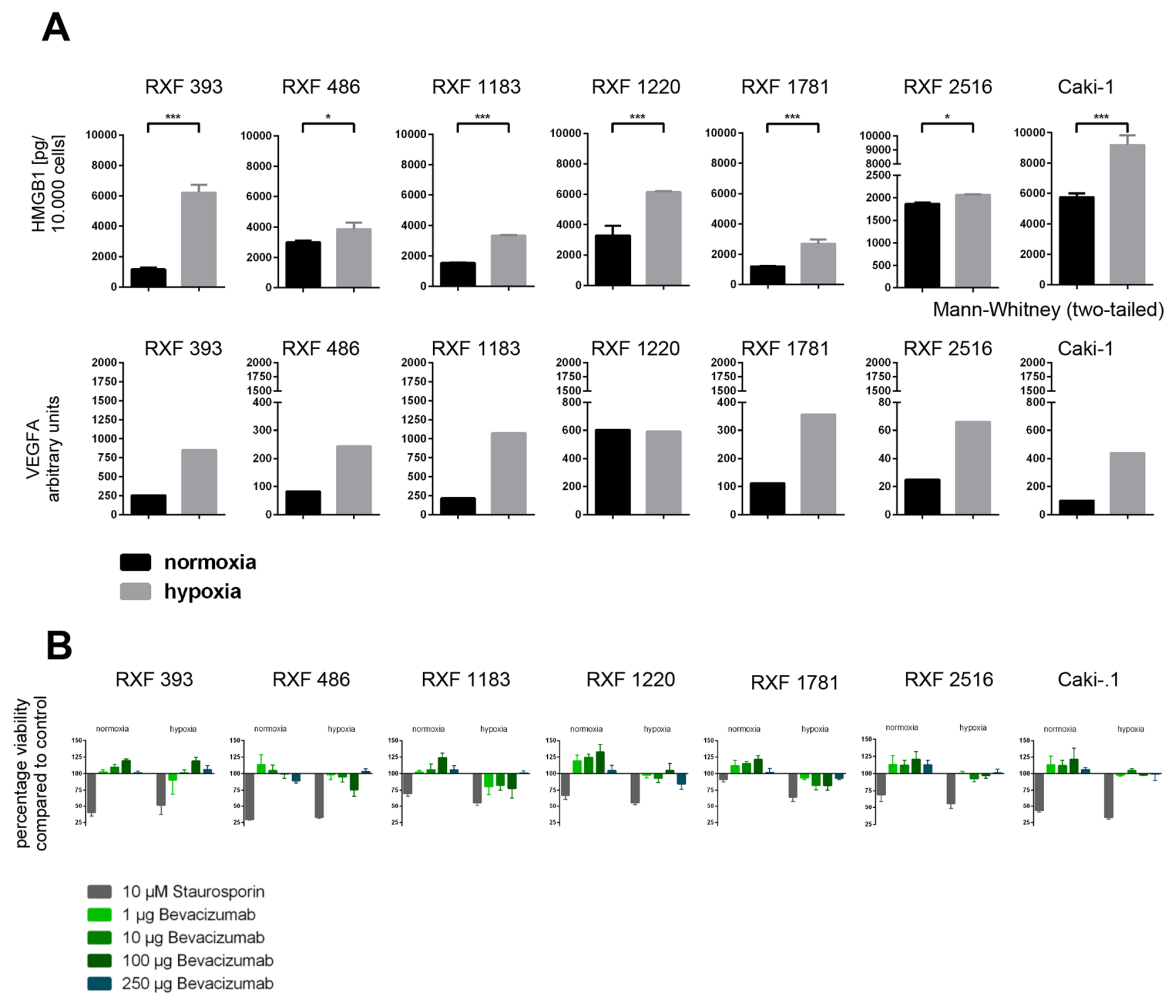
Characterization of a RCC PDX derived cell line panel under normoxic and hypoxic conditions *in vitro* in 2D **(A)** A panel of six PDX derived and one commercially available RCC cell line were cultured under normoxic and hypoxic conditions. After 96h cells were harvested, counted and analyzed. HMGB1 protein levels were determined in the supernatant by ELISA. VEGFA RNA levels were determined by qPCR. The expression level in arbitrary units was calculated as ratio of human VEGFA vs human TBP. All cell lines showed an upregulation of HMGB1 protein in the supernatant as well as VEGFA mRNA in the tumor cells. **(B)** tumor cell viability was determined after treatment with Bevacizumab and Staurosporin (positive control) under normoxic and hypoxic conditions. In general, the sensitivity towards Bevacizumab treatment was higher under hypoxic conditions whereas culture conditions had no influence on the susceptibility towards Staurosporin.

To further elucidate a potential mechanistic relationship between hypoxia and secretion of HMGB1, we evaluated secreted HMGB1 in the supernatants of PDX-derived RCC cell lines cultured *in vitro* under normoxic or hypoxic conditions for up to 96 hours. Intracellular VEGF mRNA levels were monitored as target upregulation under different culture conditions might sensitize the tumor cells towards treatment with bevacizumab [26, 27]. In all six investigated cell lines and the positive control Caki-1, hypoxia induced upregulation of secreted HMGB1. After 96h this effect was statistically significant in all investigated lines (p ranging from <0.05 to <0.005). Similarly, VEGFA mRNA levels increased accordingly, with the exception of RXF 1220. Here, VEGFA mRNA were similar between normoxic and hypoxic conditions (Figure 5A). Hypoxia also had an impact on cell proliferation: cells divided significantly slower under hypoxic conditions (Supplementary Figure 4, p<0.027). The antitumoral activity of bevacizumab *in vitro* could be enhanced by induction of hypoxia over 72h – 96h (Figure 5B and Supplementary Table 2). Hypoxia sensitized all investigated RCC cell lines towards bevacizumab treatment whereas culture conditions had no influence on the susceptibility towards the positive control staurosporin.

### Serum HMGB1 level predicts efficacy of bevacizumab *in vivo*

**Figure 6:**
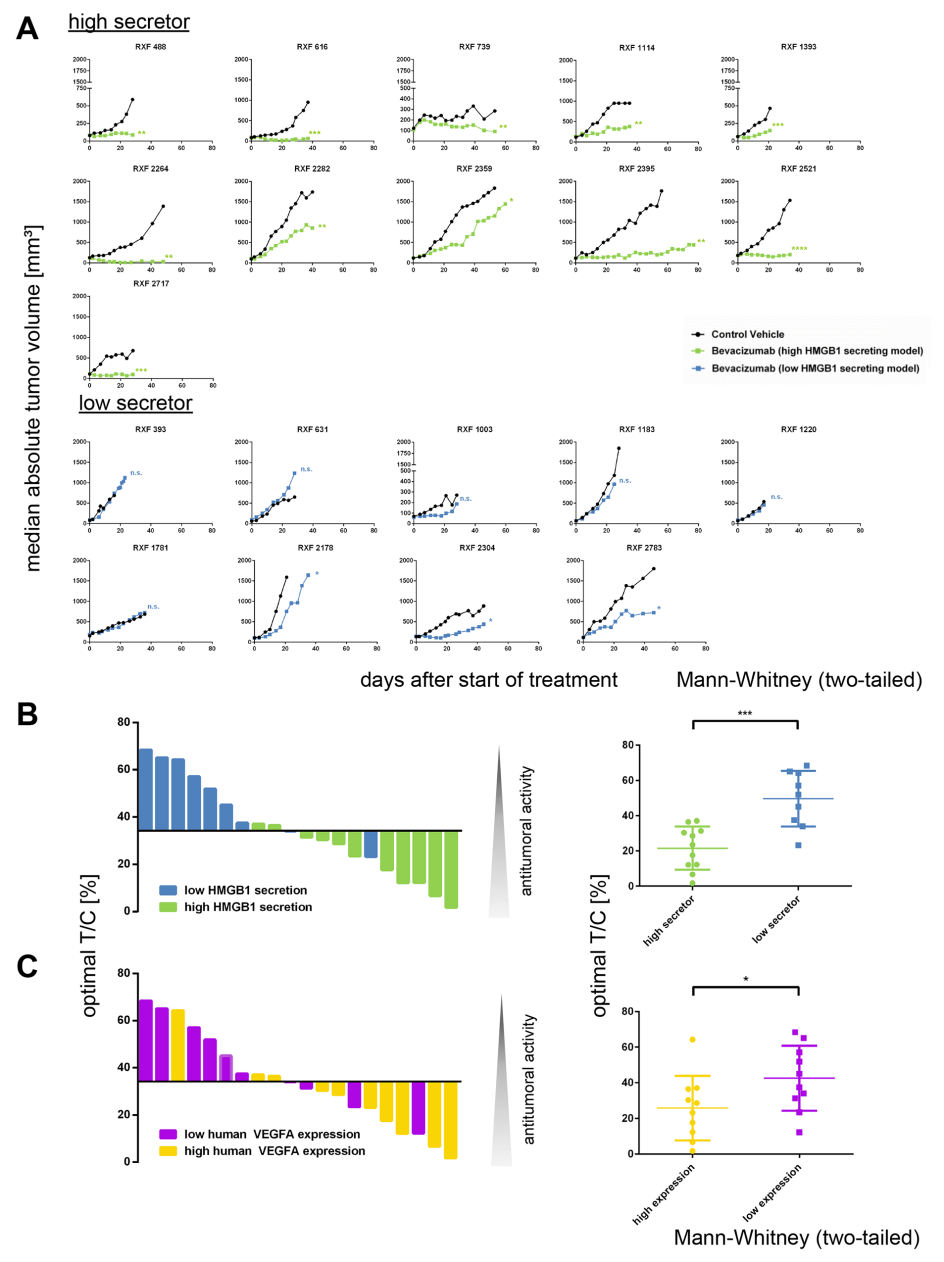
The serum level of secreted HMGB1 predicts response to treatment with bevacizumab **(A)** 20 established renal cancer PDX models were characterized bytreatment with anti-VEGF monoclonal antibody bevacizumab. Tumor volume was determined twice weekly until animals of the control group reached termination criteria. Group median absolute tumor volumes are plotted over time. Tumors with high secretion of HMGB1 (upper panel) responded significantly better to treatment with bevacizumab than tumors with low secretion of HMGB1 (lower panel). **(B)** The optimal T/C values were plotted as waterfall plot for 20 renal cancer PDX models treated with bevacizumab. The blue bars represent HMGB1 low secretor models; the green bars represent HMGB1 high secretor models. The optimal T/C values of the high secretor group were significantly lower (p < 0.0009, Mann–Whitney test, two-tailed) as the respective values of the low secretor group. **(C)** The optimal T/C values were plotted as waterfall plot for 20 renal cancer PDX models treated with bevacizumab. The purple bars represent human VEGFA low expressing models; the yellow bars represent human VEGFA high expressing models. The difference between the two groups was statically significant (p< 0.05, Mann–Whitney test, two-tailed). Thus, the level of secreted HMGB1 is a stronger predictive marker for bevacizumab sensitivity as the expression of human VEGFA.

In RCCs with high HMGB1 levels, bevacizumab induced a delay in tumor growth (RXF 1114, 1393, 2282, 2359 and 2395), stable disease (RXF 488, 739 and 2521) or even remission (RXF 616, 2264 and 2717). In contrast, within the low secretor group three tumor models depicted a moderate tumor growth delay (RXF 1003, 2178, 2304, and 2783), whereas RXF 393, 631, 1183, 1220 and 1781 were completely resistant towards treatment with bevacizumab (Figure 6A).

The correlation between sensitivity to bevacizumab and the affiliation to either low or high secretors became apparent by plotting optimal T/C values in a waterfall plot. The optimal T/C values of the high secretor group were significantly lower (p<0.0009) than the respective values of the low secretor group (Figure 6B). Among the other investigated biomarkers, only human VEGF expression in tumor tissue was also predictive for bevacizumab activity (Figure 6C, p< 0.05). Nevertheless, the level of secreted HMGB1 was a stronger predictive marker for bevacizumab sensitivity than the expression of human VEGF (Figure 6B and 6C). The combination of both biomarkers did not increase the predictive power with respect to the antitumoral activity of bevacizumab. Of note, we specifically investigated a possible link between murine VEFGA mRNA expression and bevacizumab sensitivity, but could not detect any correlation (Supplementary Figure 5, p=0.13). The observed predictivity was specific for secreted HMGB1 as well as bevacizumab: Serum HMGB1 level was exclusively predictive for bevacizumab sensitivity and not for the other investigated compounds (Mann–Whitney test).

## DISCUSSION

Considerable progress has been made in the treatment of patients with RCC. For patients with localized disease, partial nephrectomy or radical nephrectomy is standard of care depending on the size and location of the tumor. However, approximately 30% of patients present with metastatic disease at the time of diagnosis [28] or need systemic treatment for metachronous metastasis. With the introduction and approval of targeted therapies, life expectancy of patients with locally advanced and disseminated RCC has significantly improved [24]. Yet, there are currently no biomarkers available to facilitate selection of targeted tumor therapy among approved drugs for the individual patient, leaving healthcare professionals to choose at random.

In the current study, we generated advantage of a panel of PDXs and then used them to investigate biomarker candidates. We demonstrated that a subgroup of RCC PDX showed significantly elevated HMGB1 serum levels and that this easily accessible serum marker could predict response to systemic treatment with bevacizumab. 23/24 PDX were ccRCC; thus, the predictive value of HMGB1 refers to the clear cell histology of RCC. We have hereby provided evidence for a more tailored and personalized systemic therapy approach.

Preclinical tumor models are a fundamental component in the development of new anti-cancer compounds. It has been repeatedly shown that drug activity and responses in PDX models are similar to clinical therapeutic responses in patients [29–32]. They also retain the cellular and histological structure of the primary tumors. We characterized histomorphological aspects as well as the genetic landscape of our PDX panel, and confirmed the similarity of patient renal cell carcinomas and the patient-matched PDXs. Biochemical studies of the VHL signaling pathway culminated in the development of the targeted therapies [9] used in this study. pVHL status and also other known mutations involved in RCC carcinogenesis (e.g. SETD2, PBRM1, BAP1) recapitulated mutations in our PDX panel as described in the literature [8].

HMGB1 is a highly conserved chromosomal protein that functions as a chaperone in the nucleus. For example, HMGB1 enhances the function of retinoblastoma protein and prevents genomic instability [33]. HMGB1 also has a role outside the cell as a prototypical damage-associated molecular pattern that constitutes an endogenous danger signal when released by dying necrotic cells [15]. Moreover, HMGB1 is both secreted by tumor cells and found within many tumors including prostate cancer, melanoma, lung and colon cancer, and is associated with poor prognosis [34–38]. Effects of HMGB1 are mediated through various receptors including receptors for advanced glycation end products (RAGE), TLR2 and TLR4 that are expressed on endothelial cells, macrophages and several malignant cells [39]. Subsequent activation of NF-kB has been implicated in malignant cell migration and invasion [40]. mRNA expression levels of RAGE, TLR2 and TLR4 were similar in our PDX panel and did not correlate with secreted HMGB1 levels or sensitivity towards anti-angiogenic therapy (data not shown).

Especially for RCC, expression of HMGB1 was significantly higher in the tumor than in the peritumoral tissue. Furthermore, HMGB1 was associated with a lower survival rate and faster clinical progression as well as higher pathological T stages and grading [19, 41]. This correlation could not be confirmed in our PDX collection and is most likely due to the fact that late stage RCCs were overrepresented in our panel. This obvious but not strict correlation between tumor staging and engraftment capacity of the tumor tissue in mice has also been described for other types of solid cancers [42, 43]. In our study, human VEGF levels positively correlated with response to bevacizumab *in vivo*, but we determined an even stronger significant correlation between serum HMGB1 levels and response to bevacizumab. This contrasts results from another group that conducted prospective evaluation of serum VEGF levels, finding that they did not predict treatment response to bevacizumab. [44].

Apart from being released by tumor cells, HMGB1 is also secreted by macrophages and monocytes upon stimulation with cytokines in order to promote immunogenic responses [45]. RCC is frequently infiltrated with immune cells and is considered an immunogenic cancer [46]. Thus, infiltrating mouse macrophages and monocytes may also contribute to elevated HMGB1 levels, as these animals exclusively lack T and B cells yet their monocytes are largely functional. Immunohistochemical analysis in our study indicated that tumors expressed human HMGB1 protein and released it into the serum, although we did not see a correlation between secreted serum levels and intensity of DAB staining. Overall, several mechanisms are described that explain discordance between intra- and extracellular HMGB1 levels, and it is currently not completely clear if measured serum HMGB1 levels are derived from a release through tumor cells or infiltrating immune cells from the tumor microenvironment. Several mechanisms might have a modulatory effect on both the local inflammatory response at the tumor site and the rate of tumor cell growth [47].

HMGB1 induces a proangiogenic phenotype in endothelial cells [48, 49] and through their activation significantly increases the number of vessels [50]. This leads to improved vascularization of the tumor and complements the effect of the VHL mutation, which results in subsequent increased expression of hypoxia inducible factors, both resulting in tumor growth. Intratumoral hypoxia is known to be a trigger for HMGB1 translocation and release [27, 51, 33] and increased expression of HMGB1 receptors [40] Our data support a potential mechanistic relationship between VHL/hypoxia signaling and HMGB1, as VHL mutation in the PDX panel was associated with higher HMGB1 secretion in tumor-bearing mice. Hypoxia inducible factors in RCC are upregulated due to VHL mutation but also due to tumor hypoxia. Indeed, PDX-derived cell lines exhibited enhanced secretion of HMGB1 under hypoxic conditions *in vitro*. Furthermore, HIF-1 alpha expression in the PDX panel correlated significantly with serum HMGB1 levels. Accordingly, VHL downstream hypoxia signaling and tumor hypoxia might both trigger increased HMGB1 secretion, and result in increased VEGF signaling as well as total VEGF levels [52] that bevacizumab can target. However, as we did not see concomitant upregulation of VEGFA mRNA levels in all tumors, there might be also an independent pathway linking HMGB1 secretion to bevacizumab sensitivity, as previously reported [26]. The exact mechanism between tumor hypoxia signaling and intra-/extracellular HMGB1 levels needs to be further examined.

In summary, we show that serum levels of HMGB1 could predict treatment response of RCC xenografts to bevacizumab *in vivo*. Our data indicate that HMGB1 is secreted by tumor cells, but additional release through immune cells within the tumor microenvironment may contribute to elevated HMGB1 levels. Expression of HMGB1 is upregulated in many cancer types and there are preliminary data available that HMGB1 enhances tumor growth. And inhibition of the HMGB1-RAGE pathway leads to tumor growth suppression [36]. Therefore inhibition of the HMGB1–RAGE interaction might be a promising therapeutic approach for the modulation of the inflammatory and tumor-facilitating activity of HMGB1 [53].

Our findings may have direct implications for patient stratification strategies; thus, further validation of HMGB1 serum levels in patients with RCC is ongoing.

## MATERIALS AND METHODS

### Patient tumor material

Fresh sterile tumor tissue was obtained from renal cancer patients undergoing surgery at the Urology Department of the University Hospital Frankfurt and maintained in tissue transportation medium (Oncostore, Germany) at 4°C until implantation into immune compromised mice. The investigation was approved by the local ethics committee (UGO 03/13) and informed consent was obtained from the patients. Before implantation into immune deficient mice, a piece of tumor tissue was fixed in formalin and snap-frozen for subsequent analyses, respectively.

### PDX establishment

This study was carried out in strict accordance with the recommendations in the Guide for the Care and Use of Laboratory Animals of the Society of Laboratory Animals (GV SOLAS). All animal experiments were approved by the Committee on the Ethics of Animal Experiments of the regional council (Permit Numbers: G-09/58, G-13/13 and G13/43). 4- 6 week old female NMRI nu/nu mice (Charles River, Germany) placed under isoflurane anesthesia received tumor implants subcutaneously in both flanks. During the first passages, mice were monitored for tumor growth for up to 12 months. When stable tumor growth could be determined, mice were sacrificed and tumor material was implanted into new recipient mice. In addition, xenograft material was stored in liquid nitrogen for future implantation or fixed in formalin and stored liquid nitrogen for subsequent analyses. A PDX was defined as established when stable growth over at least 3 passages and regrowth from liquid nitrogen could be observed. The percentage of tumor implants displaying stable growth (take rate) and passage time were recorded for every model and every individual passage. Tumor growth was determined by a two-dimensional measurement with calipers weekly or biweekly depending on the growth characteristics of the respective PDX model. Tumor volumes were calculated according to the following equation: Tumor Vol [mm^3^] = a [mm] x b^2^ [mm^2^] x 0.5, where “a” is the largest diameter and “b” is the perpendicular diameter of the tumor representing an idealized ellipsoid. Animals had to be sacrificed when tumor volume reached 1.800 mm^3^.

### Treatment experiments *in vivo*

Implantation was performed similar to PDX establishment, except that animals received bilateral tumor implants. Animals and tumor implants were monitored daily until the maximum number of implants showed clear signs of beginning solid tumor growth. At randomization, the volume of growing tumors was initially determined. Animals bearing 50 - 250 mm^3^ tumors, preferably 80 – 200 mm^3^, were distributed into experimental groups, with comparable median and mean tumor volumes. The day of randomization was designated as day 0 of an experiment and was also the first day of dosing. All compounds were applied *via* common application routes according to the relevant animal welfare guidelines published by FELASA and GV-SOLAS. Details are listed in Supplementary Table 3 Tumor volume and body weight were determined twice weekly.

### Evaluation of anti-tumoral activity

The relative volume of an individual tumor on day X (RTV_x_) was calculated by dividing the absolute volume [mm^3^] of the respective tumor on day X (T_x_) by the absolute volume of the same tumor on the day of randomization, i. e. on day 0 (T_0_), multiplied by 100, as shown by the following equation:

$${\text{RTV}}_{\text{X}}\left[\text{\%}\right]=\frac{{\text{T}}_{\text{X}}}{{\text{T}}_{\text{0}}}\text{×}100$$

RTVs were used for compound activity rating as follows: ≤ 10 = complete remission; > 10 ≤ 50 = partial remission; > 50 ≤ 75 = minor remission; > 75 ≤ 125 = no change; > 125 progression.

Tumor inhibition on a particular day (T/C_x_) was calculated from the median RTV of a test group and the median RTV of a control group multiplied by 100, as shown by the following equation:

$${\text{T}/\text{C}}_{\text{X}}\left[\text{\%}\right]=\frac{\text{medain }{\text{RTV}}_{\text{X}}\text{ treated group}}{\text{median }{\text{TRV}}_{\text{X}}\text{ control group}}\text{×}100$$

The minimum T/C [%] value recorded for a particular group during an experiment represented the maximum anti-tumor activity for the respective compound.

### HMGB1 ELISA

The HMGB1 ELISA kit from IBL (#ST51011, Hamburg, Germany) was used according to manufacturer’s instructions. Sera from tumor-bearing mice were divided into three technical replicates. Each tumor model and time point, including non-tumor bearing mice as negative controls, comprised 3 – 5 individual mice (= biological replicates). Supernatants from 96-well culture plates were sampled at indicated time points and divided into two technical replicates. For each condition, the model and time point comprised 3-6 wells (= biological replicates).

### FFPE samples, TMA and IHC

#### FFPE

Tumors were collected immediately after euthanasia of the donor animal. FFPE fixation was performed in 10% neutral buffered formalin for 24 hours followed by routine processing and embedding into paraffin. H&E stains of all samples were performed as described [54].

#### TMA

Whole tumor sections (4 μm) were cut and stained with Hematoxylin-Eosin (H&E). H&E sections of the xenografts were studied by light microscopy and representative areas marked on the slides. Xenograft biopsies, 1 mm in diameter, were taken from the corresponding area in the paraffin block and arrayed in duplicates into a new recipient block as described [54].

#### HIF-1 alpha and HMGB1 IHC

After antigen retrieval, 5μM FFPE tissue sections were incubated with anti-human HMGB1 Antibody (1:100, Cat#3935, Cell Signaling, USA) or anti-human HIF-1 alpha Antibody (1:100, Cat#3935, Cell Signaling, USA) overnight at 4°C, followed by DAB staining and hematoxylin counterstaining.

#### Image analysis

Digitalized images of the IHC slides were evaluated to determine the percentage of HMGB1/HIF-1 alpha-positive areas using OSANO software. A computerized analysis for digitized whole-slide images of the samples was used to quantify the HMGB1/HIF1-alpha expression using color classification and morphological image processing techniques.

### qRT-PCR

#### RNA isolation

Total RNA was isolated from frozen tumor samples with the “mirVana miRNA Isolation kit” (Ambion, Carlsbad, CA, USA) according to the manufacturer’s instructions. The genomic DNA was digested using “RNase-free DNase Set” (Qiagen, Hilden, Germany). The quantity, quality and integrity of the RNA preparations were controlled using Nanodrop (Thermo Fisher Scientific, Wilmington, DE, USA) and the Bioanalyzer (Agilent Technologies, Palo Alto, CA, USA). Only RNA with 260/280 and 260/230 ratios close to 2.0 and a RIN (RNA integrity number) higher than 6.5 were used for subsequent analysis.

#### qRT-PCR

1 μg RNA was reverse transcribed into cDNA using MMLV reverse transcriptase. The resulting cDNA were analyzed in 40 cycles on a StepOnePlus™ (Applied Biosystems) using 2X KAPATM SYBR Green Fast qPCR kits (KAPA Biosystems) following the manufacturer’s recommendations. Human VEGFA, mouse VEGFA (vascular endothelial growth factor A) and human HMGB1 (High mobility group box 1) mRNA expression were determined using species-specific qRT-PCR primers (Supplementary Table 4). Furthermore, the mouse stromal content of the PDX models was quantified by mRNA expression of human and mouse TBP (TATA box-binding protein) [5, 6]. QRT-PCR of 18S ribosomal RNA (gene RNA18S1) was performed for normalization: gene of interest exp= 2^(Ct 18s – Ct gene of interest) expressed in arbitrary units (AU). The percentage of mouse RNA was obtained by dividing the calculated mouse TBP mRNA expression by the sum of human and mouse TBP mRNA expression, after normalization with 18s as described above, and multiplied by 100.

### Sequencing data acquisition

Exonic regions from PDX DNA samples were targeted using Agilent SureSelect Human All Exon kits V1 38MB, V4 51MB, V5 50MB or V6 60MB. Enriched genomic DNA was sequenced with Illumina HiSeq-2000/2500/4000 in 100bp or 126pb paired-end (PE) reads and an expected coverage on targets of ∼100X. Raw reads were subjected to fastQC to calculate read quality metrics. After alignment to the Human reference genome and Mouse reads removal, the quality of BAM files was assessed by Qualimap to obtain the percentage of mapped reads and coverage of reads to the targeted exons (as defined by Agilent). Variant detection analysis was QC-evaluated with SnpEff by computing and validating the transition/transversion ratio from SNPs found in exons.

### Statistical analysis

For the evaluation of the statistical significance of antitumor efficacy, the non-parametric Kruskal-Wallis test followed by Dunn’s method for pairwise comparisons were performed. Individual RTVs of test and control groups were compared on days on which the minimum T/C values were achieved in the test groups. For statistical analysis of survival data, the Log-rank (Mantel-Cox) test was applied. For the evaluation of the statistical significance in all other cases, the Mann–Whitney test (two-tailed) was used. By convention, p-values ≤ 0.05 indicate significant differences. Statistical calculations were performed using GraphPad Prism bio-analytic software (version 6.02 for Windows, GraphPad Software, San Diego California USA, www.graphpad.com).

### Renal cancer patient survival data

TCGA data from 505 renal cancer patients were analyzed using the ProgeneV2 prognostic database (http://watson.compbio.iupui.edu/chirayu/proggene/database/index.php) [55]. The cohort of renal cancer patients was divided into two groups defined by the median HMGB1 gene expression, and the data were adjusted for stage.

Survival data of donor patients were accessible for 25 PDX models. The cohort was divided into high and low secretors as determined by the corresponding PDX, and overall survival was compared by applying the Log-rank (Mantel-Cox) test.

### Cell culture experiments

#### Cell line establishment

Single cell suspensions from tumor tissue from established PDX were prepared as described [8] and cells were cultured in RPMI 1640 medium 2ed with 10% (vol/vol) fetal bovine serum, 1% (vol/vol) Gentamycin and 1% (vol/vol) L-glutamine (all from GIBCO-BRL, Grand Island, NY, USA). Cells were maintained at 37°C and 5% CO_2_. Cells were kept in serial passage until all mouse cells were depleted and a stable line was established (passage number >20). A STR-analysis was performed to confirm human origin and to prove conformity with the donor PDX. Caki-1 cells were sourced from ATCC and maintained under the same conditions as the PDX derived cell lines.

#### 2D cell culture under normoxic and hypoxic conditions

PDX-derived RCC cell lines were cultured under normoxic (37°C and20% O_2_) and hypoxic (37°C and 2% O2, HERACELL VIOS, tri-gas system, ThermoFisher) conditions. HMGB1 protein levels in supernatants, VEGFA mRNA expression in the tumor cells and tumor cell numbers were determined 10h, 24h, 48h, 72h and 96h after seeding. Treatment with bevacizumab was performed at 4 different dose levels (1μg, 10μg, 100μg and 250 μg/ml) over 4 days. Cells were either kept under normoxic or hypoxic conditions for the duration of the experiment. Pre-conditioning time was adapted to growth behavior of the individual cell line (for details see Supplementary Table 2). Cell viability was determined at the end of the treatment period using the cell titer blue assay (Promega, Madison, USA). 10 μM Staurosporin served as positive control and DMSO as negative controls. All experiments were performed in triplicate on 96well plates. Antiproliferative activity of the different treatments were calculated as fold-changes versus the DMSO treated control.

## SUPPLEMENTARY MATERIALS FIGURES AND TABLES




