# Supplementary material for: Patient derived renal cell carcinoma xenografts exhibit distinct sensitivity patterns in response to antiangiogenic therapy and constitute a suitable tool for biomarker development
Source: Oncotarget. 2018 Jul 24;9(57):30946–61. doi: 10.18632/oncotarget.25697 (PMC6089561; doi:10.18632/oncotarget.25697)
Supplement: Supplementary file 1 [file oncotarget-09-30946-s001.pdf]

Patient derived renal cell carcinoma xenografts exhibit distinct sensitivity patterns in response to antiangiogenic therapy and constitute a suitable tool for biomarker development

SUPPLEMENTARY MATERIALS

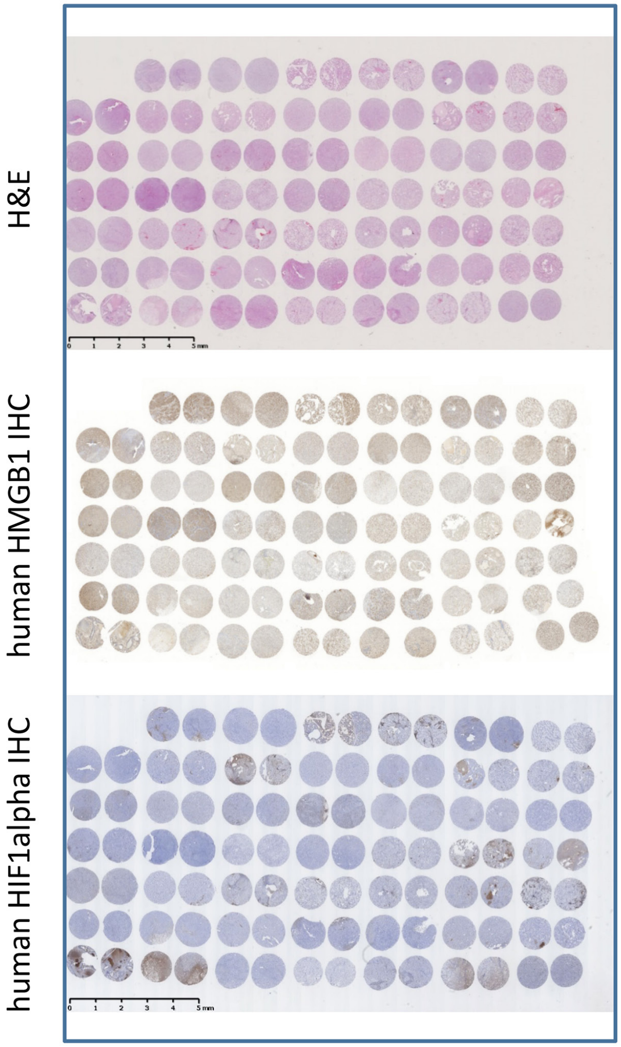

		MRI-H-166	MRI-H-166	CAKI	CAKI	RXF 2830	RXF 2830	RXF 2829	RXF 2829	RXF 2806	RXF 2806	RXF 2796	RXF 2796
RXF 2783	RXF 2783	RXF 2773	RXF 2773	RXF 2765	RXF 2765	RXF 2755	RXF 2755	RXF 2720	RXF 2720	RXF 2717	RXF 2717	RXF 2706	RXF 2706
RXF 2678	RXF 2678	RXF 2667	RXF 2667	RXF 2662	RXF 2662	RXF 2579	RXF 2579	RXF 2543	RXF 2543	RXF 2540	RXF 2540	RXF 2527	RXF 2527
RXF 2521	RXF 2521	RXF 2516	RXF 2516	RXF 2503	RXF 2503	RXF 2502	RXF 2502	RXF 2453	RXF 2453	RXF 2404	RXF 2404	RXF 2395	RXF 2395
RXF 2372	RXF 2372	RXF 2359	RXF 2359	RXF 2357	RXF 2357	RXF 2304	RXF 2304	RXF 2282	RXF 2282	RXF 2264	RXF 2264	RXF 2258	RXF 2258
RXF 2178	RXF 2178	RXF 1781	RXF 1781	RXF 1393	RXF 1393	RXF 1220	RXF 1220	RXF 1183	RXF 1183	RXF 1114	RXF 1114	RXF 1003	RXF 1003
RXF 739	RXF 739	RXF 631	RXF 631	RXF 616	RXF 616	RXF 488	RXF 488	RXF 486	RXF 486	RXF 423	RXF 423	RXF 393	RXF 393

Supplementary Figure 1: A TMA was prepared representing 44 established renal cancer PDX models in duplicate and two additional renal cancer cell line derived xenografts (Caki1 and MRI-H-166). Representatives of all three histological subtypes - clear cell, papillary and chromophobe carcinoma - were included. H&E, HMGB1 and HIF-1 alpha IHC are shown, as well as the detailed layout.

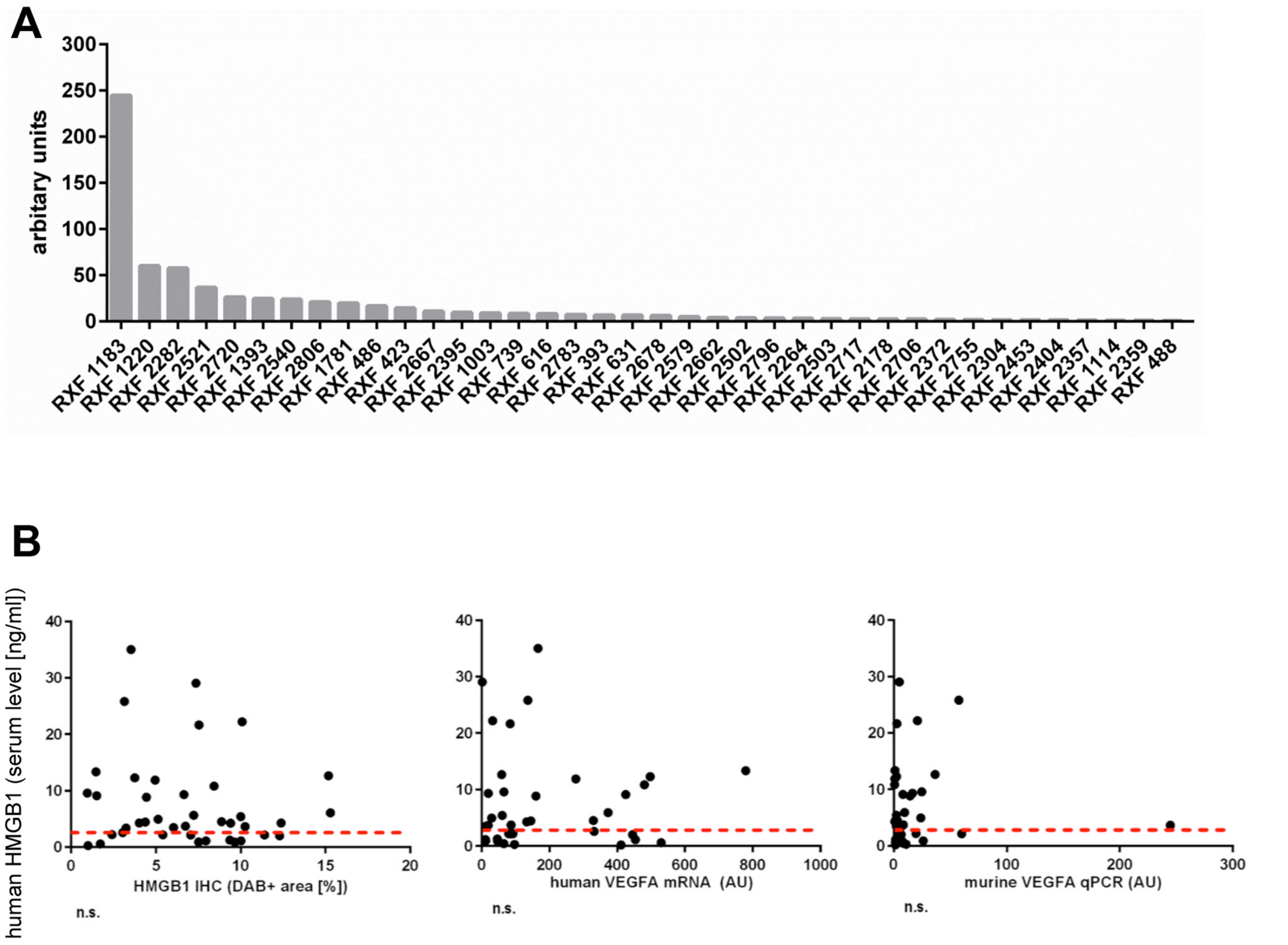

Supplementary Figure 2: **(A)** mRNA expression of mouse VEGFA determined by qPCR on lysates of renal cancer PDX tissue. The expression level in arbitrary units is calculated as the ratio of mouse VEGF to mouse TBP. **(B)** Serum levels of HMGB1 and levels of HMGB1 determined by IHC, or human VEGFA as well as murine VEGFA expression determined by qRT-PCR did not correlate. The high secretors (above the red dotted line) exhibited all different levels of IHC staining intensity and qRT-PCR expression, respectively.

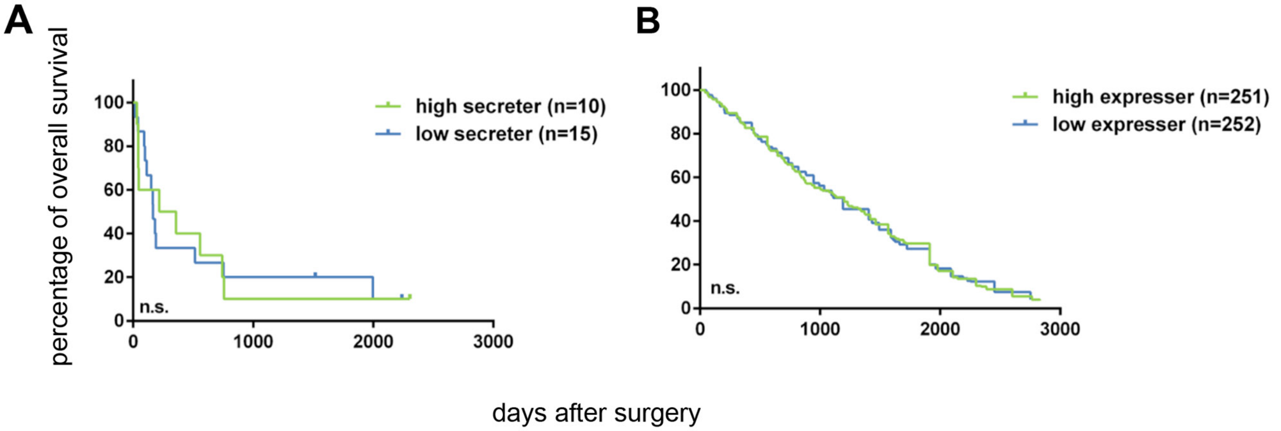

Supplementary Figure 3: **(A)** Survival data from donor patients were accessible for 25 PDX models. The cohort was divided into high and low HMGB1 secretors, as determined from their corresponding PDX models. No significant difference between the two cohorts could be detected. **(B)** TCGA data from 505 renal cancer patients were analyzed. The cohort of renal cancer patients was divided by the median of HMGB1 gene expression and the data adjusted for stage. The two groups showed no significant difference in overall survival. Taken together, HMGB1 does not appear to be a prognostic marker for overall survival.

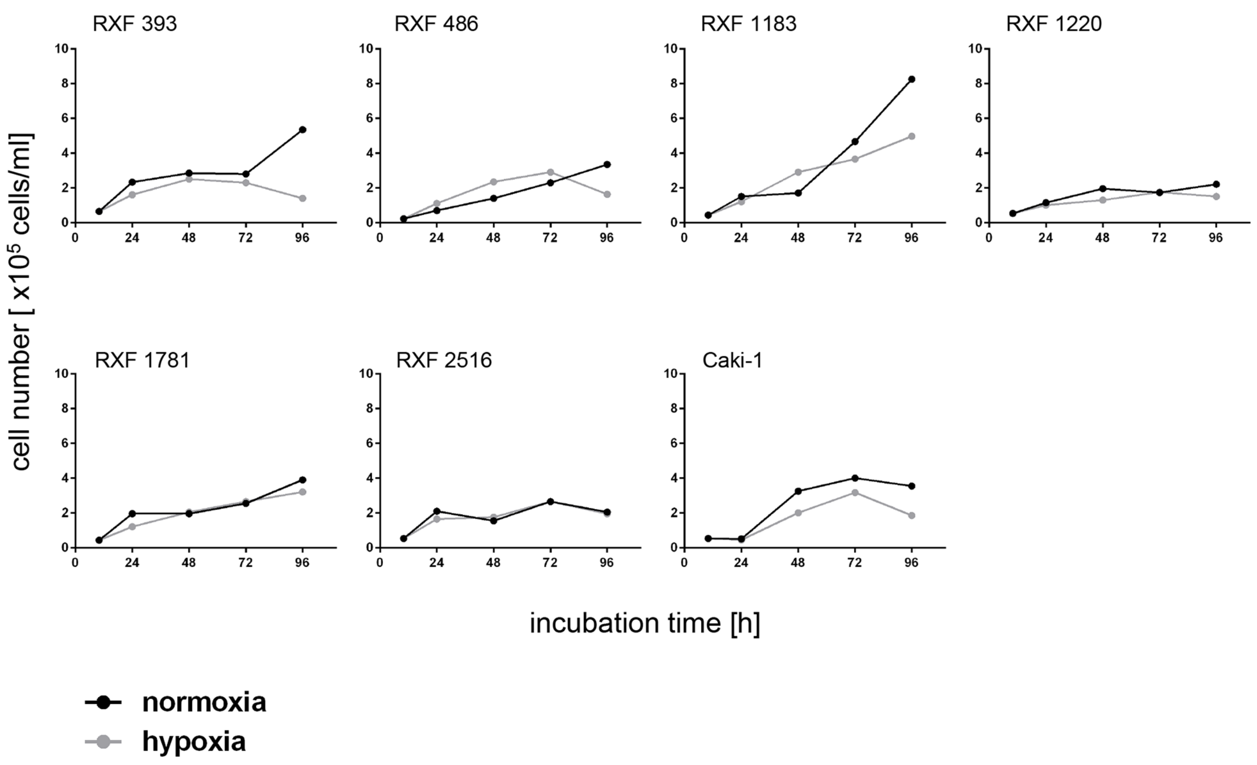

Supplementary Figure 4: A panel of six PDX derived and one commercially available RCC cell line were cultured under normoxic and hypoxic conditions. 10h, 24h, 48h, 72h and 96h after seeding cell numbers per ml were determined. The reduction of O_2 markedly reduced tumor cell proliferation in four out of seven investigated lines.

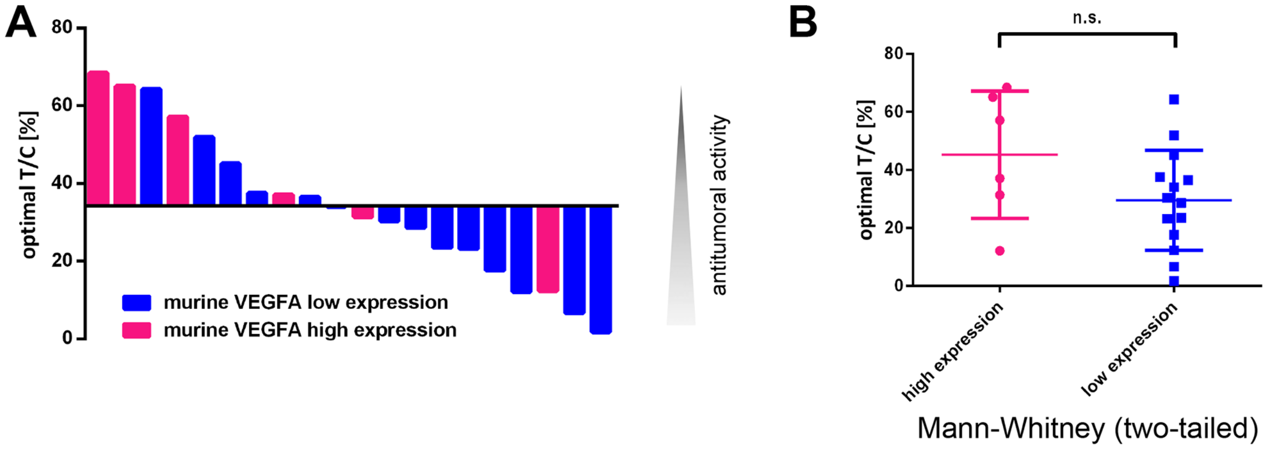

Supplementary Figure 5: **(A)** The optimal T/C values were plotted as waterfall plot for 20 renal cancer PDX models treated with bevacizumab. The dark blue bars represent mouse VEGFA-low expressing models (n = 14); the red bars represent mouse VEGFA-high expressing models (n = 6). **(B)** The difference between the T/C values of the groups was not statistically significant (Mann-Whitney test).

Supplementary Table 1: Selected mutations determined by whole exome sequencing in 39 human renal cancer PDX models.

See Supplementary File 1

Supplementary Table 2: Treatment of RCC PDX-derived cell lines with Bevacizumab under Normoxia and Hypoxia

-96 h	-72 h	0h	24h- 120h	120h
Induction of hypoxia	Induction of hypoxia	Seeding in 96-well plates	Treatment with Bevacizumab, DMSO or Staurosporin	Cell titer blue
RXF 486	RXF 1781			
RXF 1183	RXF 2282			
RXF 1220	RXF 2516			
	RXF 393			
	RXF			
	Caki1			

Supplementary Table 3: Treatment regimen of PDX-bearing immune compromised mice

Treatment	Daily dose [mg/kg]	Schedule	Application route
axitinib	25	Twice daily until end of experiment	po
bevacizumab	40/20	Once weekly	iv
pazopanib	60	Daily until end of experiment	po
sorafenib	200	Daily until end of experiment	po
sunitinib	40	Daily until end of experiment	po
temsirolimus	100	Five consecutive days	iv

po: per oral
iv: intravenous injection

Supplementary Table 4: Primers for qRT-PCR analyses

Species	Official Gene Symbol	Gene Title	Primer F sequence (5'-3')	Primer R sequence (5'-3')	Amplicon Size (bp)
human	HMGB1	High-mobility group box 1	TGATCGTCCCATCACAGTGT	TCCTACAATGTCTGAGCAATGG	213
human	VEGFA	vascular endothelial growth factor A	GGGCAGAATCATCACGAAGTG	GGTCTCGATTGGATGGCAGTA	72
mouse	VEGFA	vascular endothelial growth factor A	CGGGATTGCACGGAAACTT	GCGCAGACCACGGCTACTAC	69
human	TBP	TATA box binding protein	GGCCGCCGGCTGTT	GCTGGGTCACTGCAAAGATCA	60
mouse	TBP	TATA box binding protein	GGCGGTTTGGCTAGGTTT	GGGTTATCTTCACACACCATGA	83
human & mouse	RNA18S	Ribosomal RNA 18S	CTACCACATCCAAGGAAGGCA	TTTTTCGTCACTACCTCCCCG	71
