# Supplementary material for: Patient derived renal cell carcinoma xenografts exhibit distinct sensitivity patterns in response to antiangiogenic therapy and constitute a suitable tool for biomarker development
Source: Oncotarget. 2018 Jul 24;9(57):30946–61. doi: 10.18632/oncotarget.25697 (PMC6089561; doi:10.18632/oncotarget.25697)
Supplement: Supplementary file 2 [file oncotarget-09-30946-s002.docx]

**Supplementary Table 1: Selected mutations determined by whole exome sequencing in 39 human renal cancer PDX models**

KIRC Kidney renal clear cell carcinoma 42

KIRP Kidney renal papillary cell carcinoma 1

chromophobe RC Chromophobe renal cancer 1
